# Prevalence and genetic diversity of coronaviruses in wild birds, Finland

**DOI:** 10.1080/20008686.2017.1408360

**Published:** 2017-11-28

**Authors:** Satu Hepojoki, Erika Lindh, Olli Vapalahti, Anita Huovilainen

**Affiliations:** a Virology, University of Helsinki, Helsinki, Finland; b Helsinki University Hospital, Helsinki, Finland; c Veterinary Biosciences, University of Helsinki, Helsinki, Finland; d Research and Laboratory Department, Veterinary Virology, Finnish Food Safety Authority, Evira, Helsinki, Finland

**Keywords:** Deltacoronavirus, gammacoronavirus, wild birds, zoonoses

## Abstract

**Introduction:** Migratory birds act as hosts for a number of zoonotic viruses, and have the ability to disperse these viruses to distant geographic locations. Coronaviruses (CoVs) represent a family of zoonotic viruses with wide variety of animal hosts, including birds and humans. The infections caused by coronaviruses vary from mild to severe, depending on the viral species and the host. Since the coronaviruses exhibit extraordinary large RNA genome, also the rate of homologous recombination is high, which in turn contributes to the genetic diversity and interspecies host-switches of CoVs. The emergence of novel CoVs has been rich during the last decades, and wild birds seem to serve as reservoirs for a variety of CoV strains. We examined the CoVs circulating among wild birds in Finland.

**Materials and methods:** Samples (cloacal swab, tracheal swab, oropharyngeal swab, or tissue) representing 61 bird species were collected during 2010-2013, and examined by RT-PCR targeting the RdRp gene for the presence of CoV RNA.

**Results:** Altogether 51/939 (5.4%) of the examined birds were found positive by RT-PCR. Diverse gamma- and deltacoronavirus sequences were detected.

**Discussion:** Gamma- and deltacoronaviruses circulate among wild birds in Finland. The number of CoV-positive birds detected each year varies greatly.

## Introduction

Coronaviruses (CoVs) comprise a family under the order *Nidovirales* (family *Coronaviridae*) and infect a wide variety of mammals and birds. The course of infection varies greatly from asymptomatic to severe disease, depending on the host and virus species in question. The genome of CoVs is one of the largest (25–32 kb) viral RNA-genomes [1]. Based on phylogenetic analysis, the CoVs are divided into four different genera: *Alpha-, Beta-, Gamma*-, and *Deltacoronavirus*. The alpha- and betacoronaviruses are carried by mammals, whereas the gamma- and deltacoronaviruses mainly infect birds, with few exceptions [2,3]. The large genomes, infidelity of the RNA-dependent RNA polymerase, and high frequency of homologous RNA recombination are the main factors contributing to the high genetic diversity of CoVs [4–6].

The first CoV, Infectious bronchitis virus (IBV), was identified in 1937 [7]. IBV mainly infects chickens, but may infect other bird species as well. IBV is highly contagious and affects the respiratory tract, gut, kidney, and reproductive systems, causing substantial economic losses in the poultry industry [8]. Despite the global distribution of IBV, poultry in Finland remained free of clinical cases until April 2011 [9] after which outbreaks involving several CoV genotypes have occurred in Southern Finland.

The first human CoVs were identified in 1960s [10–12]. The human CoVs cause generally mild to moderate upper respiratory tract infections [13–15]. In 2003, a novel highly pathogenic betacoronavirus emerged in China, causing severe disease characterized by acute respiratory distress and it became known as severe acute respiratory syndrome (SARS)-CoV. The emergence of SARS-CoV [16] inspired virologists to more explore the highly divergent group of coronaviruses and their hosts, leading to the identification of a rapidly growing number of CoV species particularly in bats [2]. More recently, another highly pathogenic betaCoV infecting humans, the Middle East Respiratory Syndrome (MERS) CoV emerged in 2012 with a case fatality rate of over 40% [17,18].

Migratory birds have the ability to facilitate the dispersion of microorganisms with zoonotic potential. Wild birds have been associated with the ecology and dispersal of at least West Nile virus, tick-borne encephalitis virus, influenza A virus (IAV) and Newcastle disease virus (NDV) [19–21]. Since the discovery of IBV in 1937, it remained the only known *Gammacoronavirus* for over 50 years, but the number has increased dramatically during the last 10 years [5]. Thereafter, representatives of the genera *Gamma*- and *Deltacoronavirus* have been isolated from both wild and domestic birds including species from the order *Anseriformes, Pelecaniformes, Ciconiiformes, Galliformes, Columbiformes*, and *Charadriiformes* [22–24]. In this report we provide a description of CoV species circulating in wild birds in Finland. Altogether 939 samples representing 61 different bird species were collected during 2010–2013 and examined for the presence of CoV RNA.

## Materials and methods

### Ethical statement

All active and passive surveillance samples tested in this study, including samples provided by hunters, and injured or diseased birds were initially sent to the Finnish Food Safety Authority Evira (Helsinki, Finland) for avian influenza testing as part of a national surveillance program. All hunters had appropriate permits, and no birds were killed for research purposes. The hunted birds were shot during the annual duck-hunting season, from non-endangered species, and thereby do not require ethical approval.

### Sampling

The samples were collected as part of a national active and passive surveillance program, coordinated by the Finnish Food Safety Authority Evira and University of Helsinki. The sample panels included (1) hunted clinically healthy birds (active surveillance), (2) hunted clinically diseased birds (active surveillance), and (3) birds found dead (passive surveillance) or (4) clinically diseased (passive surveillance) (Table S1). The sample material used for RNA detection was cloacal swab, tracheal swab, oropharyngeal swab, or tissue. The swab samples were collected using nylon swabs and stored and transported in Universal Transport Medium (both by Copan International). Prior to RNA isolation from the swab specimen (stored at −80°C), the samples were centrifuged to remove solid particles and supplemented with additional antibiotics (streptomycin-penicillin). The tissue samples were homogenized and separated by centrifugation and supplied with antibiotics (streptomycin-penicillin). The sample panel from 2010 consisted of 343 samples, the 2011 panel of 171 samples, the 2012 panel of 287 samples, and the 2013 panel of 138 samples. The samples were also screened for influenza A virus RNA [25].

### Detection of coronavirus RNA

QIAamp Viral RNA Mini Kit was used to extract the RNA from the samples. The CoV screening was performed using degenerate primers [26] to amplify a 179 nt region in the polymerase (RdRp) gene (Orf1b). All positive samples were further characterized by amplifying a longer (608–610 bp) region in the RdRp gene using previously described primers (forward primer 5ʹ-TGGGWTGGGAYTAYCCWAARTGYGA-3ʹ and reverse primer 5ʹ-GCATWGTRTGYTGNGARCARAATTC-3ʹ) [23]. All PCR reactions were performed on a BioRad PTC-100 Thermal cycler or Applied Biosystems Veriti Thermal cycler by a one-step RT-PCR protocol using QIAGEN One-Step RT-PCR Kit (Qiagen, Germany). The reaction volume for PCR was 25 µl containing 5 µl 5× buffer, 1 µl dNTP mix, 1 µM both primers, 0.2 µl RNAse inhibitor (Applied Biosystems, 20 U/µl), 2 µl RNA, and 1 µl Qiagen One-Step RT-PCR Enzyme Mix with thermal conditions as follows: reverse transcription at 50°C for 30 min, initial PCR activation at 95°C for 15 min, followed by denaturation step at for 30 s, annealing at 50°C for 30 s, and extension at 72°C for 30 s. The PCR products were analyzed on 2% agarose gels and gel purified using QIAquick gel extraction kit (Qiagen).

### Sequencing and phylogenetic analysis

The RT-PCR amplicons were sequenced in both directions at the Sequencing Unit of Institute for Molecular Medicine Finland, (Helsinki), using BigDye v3.1 chemistry and run by ABI3730xl DNA Analyzer. The sequences were assembled in BioEdit and aligned using ClustalW [27–29]. The reference sequences were obtained through BLAST search [30]. and from GenBank [31]. Phylogenetic analyses were based on sequence alignments of 464 nucleotides (position 100–547 of the RdRp gene, by IBV (AY392086) count) within Orf1ab. Sequence data from 46 of our samples was included in the analysis and the rest were left out because of inadequate sequence quality. The phylogenetic tree was constructed from Maximum-likelihood phylogenies and bootstrap values calculated by 1000 replicates. Gaps were handled by partial deletion. The evolutionary model GTR + G + I was used after evaluation of the best-fit model according to Bayesian Information Criterion. The alignments, selection of evolutionary model and phylogenetic analyses were performed within Mega5 [29].

## Results

### Coronavirus RNA in wild birds

**Figure 1.**
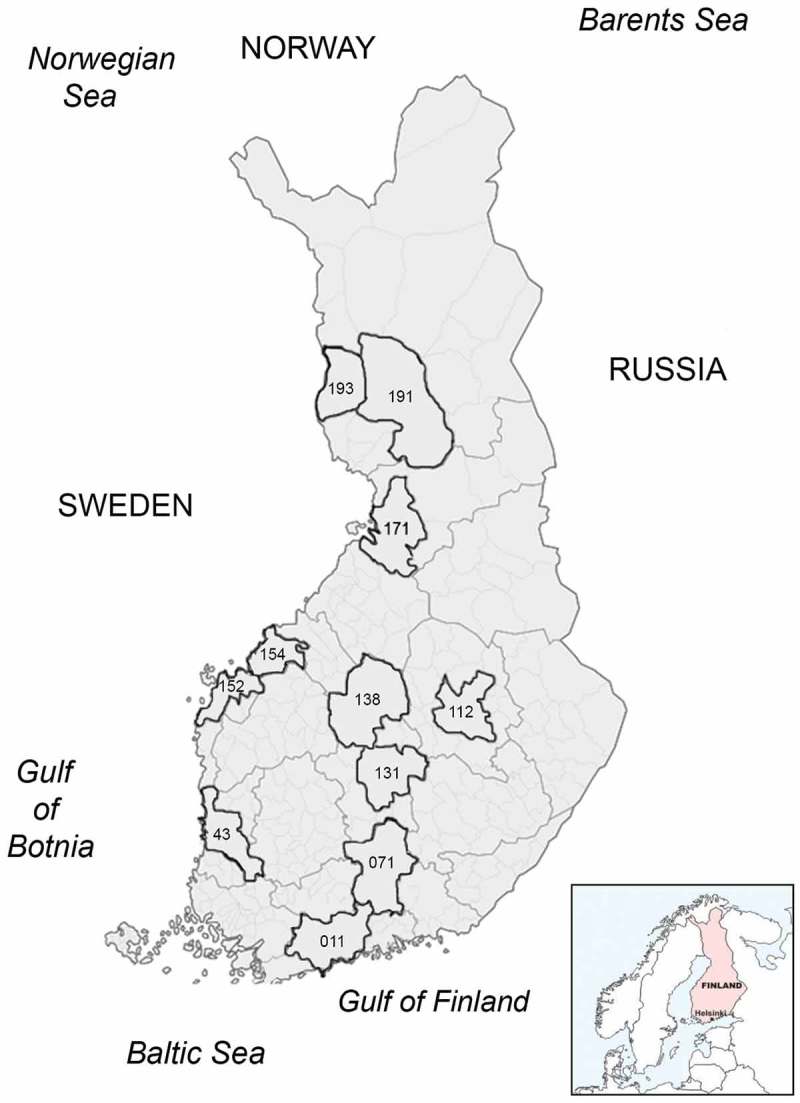
A map illustrating the sampling locations. The sampling locations (sub-regional units, year 2013) are indicated in the Table 2, and are here illustrated by numbers on a map of Finland.

We screened in total 939 samples from 61 bird species (Table S1) during a time period of four years (2010–2013) using a traditional, conserved RT-PCR targeting a 179 fragment of the RdRp gene (Orf1ab) of all coronavirus lineages. Altogether 51/939 (5.4%) of the examined birds were found to be CoV RNA positive (Tables 1 and 2), of which 27 were found healthy, and 24 dead or diseased. CoV RNA was detected in eight species (*Anas platyrhynchos, Anas crecca, Clangula hyemalis, Cygnus cygnus, Larus argentatus, Chroicocephalus ridibundus, Larus fuscus*, and *Columpa sp*.). CoV RNA was most abundant in the samples from 2010 (11%) and 2013 (7.2%), whereas during 2011 (0%) and 2012 (0.7%) only few of the tested samples were found positive. Influenza A virus (IAV) was also detected in four of the CoV positive samples (Table 1) [25,32]. The sampling locations are illustrated in Figure 1.

**Table 1.** Bird samples positive for coronavirus RNA.

Species	Clinical status (no.)	% (no. positive/negative)	Gamma	Delta	Co-infections (no.)
*Anas platyrhynchos*	Hunted clinically healthy (27) and hunted clinically diseased (1)	21.7% (28/129)	x		H9N2 (1) + H3N8 (2)
*Anas crecca*	Hunted clinically healthy	16.4% (9/55)	x		H3N8 (1)
*Larus argentatus*	Found dead	9.6% (5/52)	x		
*Chroicocephalus ridibundus*	Found dead	4.2% (1/24)		x	
*Larus fuscus*	Found dead	8.3% (1/12)		x	
*Columba sp.*	Hunted clinically diseased (1) and found clinically diseased (1)	3.6% (2/56)	x		
*Cygnus cygnus*	Found dead	3.6% (1/78)	x		
*Clangula hyemalis*	Found dead	1.3% (4/8)	x		
Total		51/412

**Table 2.** Avian coronaviruses described in this study. The strain names, species, clinical status of the bird, date of sampling, location of the sample indicated in the codes of sub-regional units of Finland, and Genbank accession numbers.

Taxonomic Family of the host	Strain	Species	Clinical status*	Date of sampling	Location	GenBank accession number
Anatidae	Avian Coronavirus/*Anas platyrhynchos*/Finland/8585/2010	Mallard (*Anas platyrhynchos*)	1	20 August 2010	112	KX588654
	Avian Coronavirus/*Anas crecca*/Finland/8589/2010	Eurasian Teal (*Anas crecca*)	1	20 August 2010	112	KX588655
	Avian Coronavirus/*Anas platyrhynchos*/Finland/8597/2010	Mallard (*Anas platyrhynchos*)	1	20 August 2010	112	KX588657
	Avian Coronavirus/*Anas platyrhynchos*/Finland/8627/2010	Mallard (*Anas platyrhynchos*)	1	20 August 2010	112	KX588658
	Avian Coronavirus/*Anas crecca*/Finland/8629/2010	Eurasian Teal (*Anas crecca*)	1	20 August 2010	112	KX588656
	Avian Coronavirus/*Anas platyrhynchos*/Finland/8764/2010	Mallard (*Anas platyrhynchos*)	1	20 August 2010	193	KX588659
	Avian Coronavirus/*Anas crecca*/Finland/8770/2010	Eurasian Teal (*Anas crecca*)	1	20 August 2010	193	KX588660
	Avian Coronavirus/*Anas platyrhynchos*/Finland/8782/2010	Mallard (*Anas platyrhynchos*)	1	20 August 2010	193	KX588661
	Avian Coronavirus/*Anas crecca*/Finland/8788/2010	Eurasian Teal (*Anas crecca*)	1	21 August 2010	193	KX588662
	Avian Coronavirus/*Anas platyrhynchos*/Finland/8790/2010	Mallard (*Anas platyrhynchos*)	1	21 August 2010	193	KX588663
	Avian Coronavirus/*Anas platyrhynchos*/Finland/8802/2010	Mallard (*Anas platyrhynchos*)	1	20 August 2010	112	KX588652
	Avian Coronavirus/*Anas platyrhynchos*/Finland/8812/2010	Mallard (*Anas platyrhynchos*)	1	20 August 2010	112	KX588653
	Avian Coronavirus/*Anas platyrhynchos*/Finland/9135/2010	Mallard (*Anas platyrhynchos*)	1	20 August 2010	131	KX588664
	Avian Coronavirus/*Anas platyrhynchos*/Finland/9163/2010	Mallard (*Anas platyrhynchos*)	1	25 August 2010	131	KX588665
	Avian Coronavirus/*Anas crecca*/Finland/9195/2010	Eurasian Teal (*Anas crecca*)	1	28 August2010	152	NA
	Avian Coronavirus/*Anas crecca*/Finland/9203/2010	Eurasian Teal (*Anas crecca*)	1	28 August2010	152	KX588666
	Avian Coronavirus/*Anas crecca*/Finland/9209/2010	Eurasian Teal (*Anas crecca*)	1	28 August2010	152	KX588667
	Avian Coronavirus/*Larus argentatus*/Finland/9211/2010	European Herring Gull (*Larus argentatus*)	3	1 September 2010	171	KX588668
	Avian Coronavirus/*Anas platyrhynchos*/Finland/9356/2010	Mallard (*Anas platyrhynchos*)	1	24 August 2010	171	KX588669
	Avian Coronavirus/*Anas crecca*/Finland/9362/2010	Eurasian Teal (*Anas crecca*)	1	28 August 2010	171	KX588670
	Avian Coronavirus/*Anas platyrhynchos*/Finland/9368/2010	Mallard (*Anas platyrhynchos*)	1	30 August 2010	171	KX588671
	Avian Coronavirus/*Anas platyrhynchos*/Finland/10485/2010	Mallard (*Anas platyrhynchos*)	1	20 August 2010	43	KX588638
	Avian Coronavirus/*Anas crecca*/Finland/10539/2010	Eurasian Teal (*Anas crecca*)	1	28 September 2010	154	KX588629
	Avian Coronavirus/*Anas platyrhynchos*/Finland/13345/2010	Mallard (*Anas platyrhynchos*)	1	2 September 2010	71	KX588635
	Avian Coronavirus/*Anas platyrhynchos*/Finland/13347/2010	Mallard (*Anas platyrhynchos*)	1	2 September 2010	71	KX588639
	Avian Coronavirus/*Anas platyrhynchos*/Finland/13359/2010	Mallard (*Anas platyrhynchos*)	1	2 September 2010	71	KX588640
	Avian Coronavirus/*Anas platyrhynchos*/Finland/13361/2010	Mallard (*Anas platyrhynchos*)	1	2 September 2010	71	KX588641
	Avian Coronavirus/*Anas platyrhynchos*/Finland/13365/2010	Mallard (*Anas platyrhynchos*)	1	2 September 2010	71	KX588642
	Avian Coronavirus/*Anas platyrhynchos*/Finland/13367/2010	Mallard (*Anas platyrhynchos*)	1	2 September 2010	71	KX588643
	Avian Coronavirus/*Anas platyrhynchos*/Finland/13369/2010	Mallard (*Anas platyrhynchos*)	1	2 September 2010	71	KX588644
	Avian Coronavirus/*Anas platyrhynchos*/Finland/13371/2010	Mallard (*Anas platyrhynchos*)	1	2 September 2010	71	KX588645
	Avian Coronavirus/*Anas platyrhynchos*/Finland/13377/2010	Mallard (*Anas platyrhynchos*)	1	2 September 2010	71	KX588646
	Avian Coronavirus/*Anas platyrhynchos*/Finland/13385/2010	Mallard (*Anas platyrhynchos*)	1	2 September 2010	71	KX588647
	Avian Coronavirus/*Anas platyrhynchos*/Finland/13389/2010	Mallard (*Anas platyrhynchos*)	1	2 September 2010	71	KX588648
	Avian Coronavirus/*Anas platyrhynchos*/Finland/13395/2010	Mallard (*Anas platyrhynchos*)	1	2 September 2010	71	KX588649
	Avian Coronavirus/*Anas platyrhynchos*/Finland/13399/2010	Mallard (*Anas platyrhynchos*)	1	2 September 2010	71	KX588650
	Avian Coronavirus/*Anas platyrhynchos*/Finland/13401/2010	Mallard (*Anas platyrhynchos*)	1	2 September 2010	71	NA
	Avian Coronavirus/*Cygnus cygnus*/Finland/4983/2013	Whooper Swan (*Cygnus cygnus*)	3	28 July 2013	71	KX588672
	Avian Coronavirus/*Clangula hyemalis*/Finland/14383/2013	Long-tailed Duck (*Clangula hyemalis*)	3	21 October 2013	11	NA
	Avian Coronavirus/*Clangula hyemalis*/Finland/14385/2013	Long-tailed Duck (*Clangula hyemalis*)	3	21 October 2013	11	NA
	Avian Coronavirus/*Clangula hyemalis*/Finland/14387/2013	Long-tailed Duck (*Clangula hyemalis*)	3	21 October 2013	11	NA
	Avian Coronavirus/*Clangula hyemalis*/Finland/14395/2013	Long-tailed Duck (*Clangula hyemalis*)	3	6 September 2012	11	KX588651
	Avian Coronavirus/*Anas platyrhynchos/Finland/11130/2013*	Mallard (*Anas platyrhynchos*)	2	2012	138	KX588637
	Avian Coronavirus/*Larus argentatus*/Finland/9211/2010	European Herring Gull (*Larus argentatus*)	3	1 September 2010	171	NA
Laridae	Avian Coronavirus/*Larus fuscus*/Finland/10059/2013	Lesser Black-backed Gull (*Larus fuscus*)	3	29 July 2013	43	KX588673
	Avian Coronavirus/*Chroicocephalus ridibundus*/Finland/10083/2013	Black-headed Gull (*Chroicocephalus ridibundus*)	3	14 August 2013	191	KX588674
	Avian Coronavirus/*Larus argentatus*/Finland/10877/2013	European Herring Gull (*Larus argentatus*)	3	14 August 2013	171	KX588630
	Avian Coronavirus/*Larus argentatus*/Finland/10879/2013	European Herring Gull (*Larus argentatus*)	3	9 September 2013	171	KX588631
	Avian Coronavirus/*Larus argentatus*/Finland/13125/2013	European Herring Gull (*Larus argentatus*)	3	21 October 2013	171	KX588633
	Avian Coronavirus/*Larus argentatus*/Finland/12822/2012	European Herring Gull (*Larus argentatus*)	3	6 September 2012	171	KX588634
Columbidae	Avian Coronavirus/*Columba sp*./Finland/11782/2013	Pigeon (*Columba sp*.)	2	4 October 2013	11	KX588632
	Avian Coronavirus/*Columba sp*./Finland/6709/2012	Pigeon (*Columba sp*.)	4	28 July 2013	11	KX588636

*Clinical status. #1 hunted and clinically healthy. #2 hunted clinically diseased. #3 found dead. #4 found clinically diseased.

### Phylogenetic analyses of the detected avian coronaviruses

**Figure 2.**
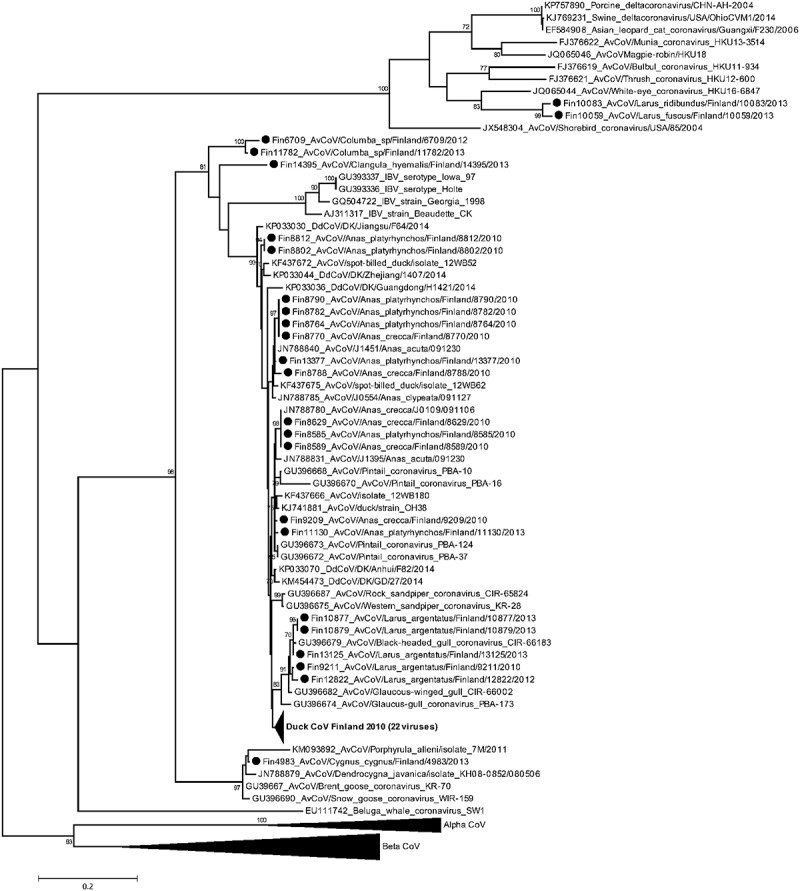
Maximum likelihood phylogeny of avian coronaviruses detected in Finland in wild birds. A 464 nucleotide long sequence region of the RdRp gene within Orf1ab was sequenced and aligned with reference sequences: the analysis includes alignments of 46 viruses detected in this study and a diverse set of reference strains obtained through BLAST search. Maximum-likelihood analysis using 1000 bootstrap replicates was used to infer tree topology. The evolutionary model GTR+G + I was used after evaluation of the best-fit model according to Bayesian Information Criterion. Bootstrap support values exceeding 70 are shown next to the nodes. Strains described in this study are marked by dots. Branches have been collapsed where our viruses share high sequence identity, and the branch wideness is relative to the number of viruses included.

The samples positive in the initial RT-PCR screen were consequently amplified for phylogenetic purposes by the method described by [23]. The number of reference strains for the alignments was unfortunately limited as GenBank deposited sequences vary in both location and length as a result of the wide array of approaches that have been employed for sequencing of coronaviruses. Table 2 shows the GenBank accession numbers of the viruses illustrated in the phylogenetic tree (Figure 2).

The viruses detected from wild ducks shared up to 99% nucleotide identity with other known CoVs of the *Gammacoronavirus* genus. The phylogenetically most closely related viruses are isolates from ducks in Russia and USA, China [33], Hong Kong [24] and South Korea [33,34]. The gammacoronaviruses that were detected in gulls in this study shared up to 99% sequence identity and clustered with previous isolates from gulls in Siberia and Alaska [23]. The viruses isolated from pigeons (*Columba* sp) (Fin6709 and Fin11782) and an isolate from a long-tailed duck (*Clangula hyemalis*) shared only up to 89% nucleotide identity with the currently published strains, with the highest nucleotide identity with duck gammacoronaviruses. Two isolates, AvCoV/*Larus fuscus*/Finland/10059/2013 and AvCoV/*Chroicocephalus ridibundus*/Finland/10083/2013, share 83% and 85% nucleotide identity with published avian and mammalian deltacoronaviruses, respectively. They clustered in the *Deltacoronavirus* clade in the ML trees, most closely to the White-eye CoV isolated in Hong Kong (JQ065044) (Figure 2). None of the viruses from wild birds in this study showed close relatedness to circulating IBV-strains.

## Discussion

Several viruses, including zoonotic and economically significant pathogens, are known to circulate among wild birds. CoVs are a large group of viruses infecting mammals and birds, including infectious bronchitis virus (IBV), which is a highly contagious coronavirus infecting chickens. A variety of coronaviruses are known to circulate among wild birds. We set up this study to determine the occurrence of coronavirus infections in wild birds in Finland. Altogether 939 samples collected between 2010 and 2013 from wild birds were included in the study. The prevalence was fairly high (up to 11%) although with great annual variation. The sample panels differed to some extent in terms of time and geographic distribution (Figure 2) and sampled species, which might partly explain the annual variation. For example, majority of the samples of 2010 were from duck species (Anas sp.) sampled for active surveillance purposes. In 2011–2013 on the contrary, most samples were derived through passive surveillance and included a smaller proportion of ducks. However, no firm conclusions can be drawn whether the observed difference in prevalence is due to some regular pattern, or due to the sampling methods (heterogeneity of the sample material).

Some of the CoV positive samples were also positive for H3N8 and H9N2 IAV RNA. Similar double infections have been reported by other researchers [35], and low pathogenic avian influenza viruses (LPAI) together with type-1 avian paramyxoviruses (APMV) are also commonly detected [36]. The co-infections observed here were in four apparently healthy ducks, three mallards and a teal, suggesting that infections by a number of low virulence viruses are well tolerated by migratory ducks.

CoV infections in mallard ducks (*Anas platyrhynchos*) have been documented in Sweden [26,35], and one of the studies reported the prevalence of CoV infections to be 6.9% albeit with seasonal variation [35]. In a more recent study, Wille et al. documented the prevalence of CoVs among wild waterbirds in Sweden to be even 18.7% [37]. A study from Norway reported the prevalence of CoV RNA among graylag goose (*Anser anser*) in Northern Europe to be as high as 38% in 2004, but they also documented great annual and geographical variation; in 2003 the prevalence was only 18% [38]. Also in England, wildfowl (*Anseriformes*) and waders (*Charadriiformes*) have been reported to carry CoVs [39]. A report from South Korea documented a prevalence of CoV RNA to be 0.95% in wildfowl (*Anseriformes*), and they also observed annual variation in the detection of CoV RNA [34]. Based on the studies from South Korea [34] and Northern Europe [38], and the results we obtained, it seems that large annual variation in the circulating RNA seems to be characteristic to avian coronaviruses. A study in China reported *Gammacoronavirus* and *Deltacoronavirus* RNA in 12% of the screened, apparently healthy, wild aquatic birds (*Anseriformes, Pelecaniformes*, and *Ciconiiformes*) [24], which is in concordance with our results. Another study, conducted in the Beringia area (encompassing areas of Alaska and Siberia), reported *Gammacoronavirus* RNA in 6.4% of the examined birds (*Anseriformes, Pelecaniformes*, and *Charadriiformes*) [23]. Interestingly, some of the sequences found in the Chinese study [24] were identical to those found from the Beringia area [23]. Also the Finnish Duck CoVs reported in this study cluster very closely with duck CoVs of the *Gammacoronavirus* genus from Siberia and China, which are connected by migratory routes. The close genetic relatedness of these strains (as well as those found in South Korea and England) indicates that migratory birds do have a specific role in dispersing CoV to distinct geographical locations. However, the sequence analyses might be somewhat biased due to the limited availability of reference strains. This and previous studies have mainly been focused on duck and shorebird species of the Northern hemisphere, hence the involvement of other avian species and a wider global distribution of avian CoVs remains unclear.

Interestingly enough our results show that the genus *Larus* (order *Charadriiformes*) can serve as a host for both genera, the *Deltacoronavirus* (species: *Larus fuscus*) and *Gammacoronavirus* (species: *Larus argenatus*). Also, the fact that we detected a *Deltacoronavirus* in *Chroicocephalus ridibundus* is interesting, since in another study [23] *Gammacoronavirus* RNA was found from the same bird species. It was previously reported, that the bird species from the order *Anseriformes* can be infected with both delta- and gammacoronaviruses [24], and we now demonstrate that the same applies for the order *Charadriiformes*. The abundance of the detected CoV sequences originated from ducks supports the hypothesis that the Duck CoVs would persist as endemic in duck populations causing subclinical infections similarly to LPAI and APMV.

To conclude, we show that there is a wide range of gammacoronaviruses and also some deltacoronaviruses circulating in wild birds in Finland. In addition, we show that the number of CoV-positive birds detected each year varies greatly. Due to the high recombination rate of CoVs, new species constantly arise that are able to adapt to new hosts and ecological niches [5]. Since the number of CoV sequences detected from birds was so abundant, it would be interesting to study what types of CoVs are circulating in small Finnish mammals, especially those in close contact with birds.

## References

[1] Flint S, Enquist L, Racaniello V, et al, editors. Principles of virology. 3rd ed. Washington (USA): ASM Press; 2008.

[2] King AMQ, Adams MJ, Carstens EB, et al, editors. Virus taxonomy: classification and nomenclature of viruses: ninth report of the international committee on taxonomy of viruses. 9th ed. San Diego (CA): Academic Press; 2012.

[3] Berson SA, Yalow RS, Bauman A, et al Insulin-I131 metabolism in human subjects: demonstration of insulin binding globulin in the circulation of insulin treated subjects. J Clin Invest. 1956;35:1–8. 13286336 10.1172/JCI103262 PMC438794

[4] Jackwood MW, Hall D, Handel A. Molecular evolution and emergence of avian gammacoronaviruses. Infect Genet Evol. 2012;12:1305–1311. 22609285 10.1016/j.meegid.2012.05.003 PMC7106068

[5] Woo PC, Lau SK, Huang Y, et al Coronavirus diversity, phylogeny and interspecies jumping. Exp Biol Med (Maywood). 2009;234:1117–1127. 19546349 10.3181/0903-MR-94

[6] Denison MR, Graham RL, Donaldson EF, et al Coronaviruses: an RNA proofreading machine regulates replication fidelity and diversity. RNA Biol. 2011;8:270–279. 21593585 10.4161/rna.8.2.15013 PMC3127101

[7] Beaudette FR, Hudson CB Cultivation of the virus of infectious bronchitis. J Am Vet Med Assoc. 1937;90:51–60.

[8] Cavanagh D Coronavirus avian infectious bronchitis virus. Vet Res. 2007;38:281–297. 17296157 10.1051/vetres:2006055

[9] Pohjola LK, Ek-Kommonen SC, Tammiranta NE, et al Emergence of avian infectious bronchitis in a non-vaccinating country. Avian Pathol. 2014;43:244–248. 24766156 10.1080/03079457.2014.913770 PMC7114077

[10] Tyrrell DA, Bynoe ML Cultivation of viruses from a high proportion of patients with colds. Lancet. 1966;1:76–77. 4158999 10.1016/s0140-6736(66)92364-6

[11] Hamre D, Procknow JJ A new virus isolated from the human respiratory tract. Proc Soc Exp Biol Med. 1966;121:190–193. 4285768 10.3181/00379727-121-30734

[12] McIntosh K, Dees JH, Becker WB, et al Recovery in tracheal organ cultures of novel viruses from patients with respiratory disease. Proc Natl Acad Sci U S A. 1967;57:933–940. 5231356 10.1073/pnas.57.4.933 PMC224637

[13] van der Hoek L, Pyrc K, Jebbink MF, et al Identification of a new human coronavirus. Nat Med. 2004;10:368–373. 15034574 10.1038/nm1024 PMC7095789

[14] Woo PC, Lau SK, Chu CM, et al Characterization and complete genome sequence of a novel coronavirus, coronavirus HKU1, from patients with pneumonia. J Virol. 2005;79:884–895. 15613317 10.1128/JVI.79.2.884-895.2005 PMC538593

[15] van der Hoek L Human coronaviruses: what do they cause? Antivir Ther. 2007;12:651–658. 17944272

[16] Drosten C, Günther S, Preiser W, et al Identification of a novel coronavirus in patients with severe acute respiratory syndrome. N Engl J Med. 2003;348:1967–1976. 12690091 10.1056/NEJMoa030747

[17] Corman VM, Eckerle I, Bleicker T, et al Detection of a novel human coronavirus by real-time reverse-transcription polymerase chain reaction. Euro Surveill. 2012;17:20285. 23041020 10.2807/ese.17.39.20285-en

[18] Cotten M, Watson SJ, Zumla AI, et al Spread, circulation, and evolution of the middle east respiratory syndrome coronavirus. MBio. 2014;5:e01062-13. 24549846 10.1128/mBio.01062-13 PMC3944817

[19] Reed KD, Meece JK, Henkel JS, et al Birds, migration and emerging zoonoses: west nile virus, lyme disease, influenza A and enteropathogens. Clin Med Res. 2003;1:5–12. 15931279 10.3121/cmr.1.1.5 PMC1069015

[20] Hubálek Z An annotated checklist of pathogenic microorganisms associated with migratory birds. J Wildl Dis. 2004;40:639–659. 15650082 10.7589/0090-3558-40.4.639

[21] Gilbert M, Xiao X, Domenech J, et al Anatidae migration in the western Palearctic and spread of highly pathogenic avian influenza H5NI virus. Emerg Infect Dis. 2006;12:1650–1656. 17283613 10.3201/eid1211.060223 PMC3372333

[22] Honkavuori KS, Briese T, Krauss S, et al Novel coronavirus and astrovirus in Delaware Bay shorebirds. PLoS One. 2014;9:e93395. 24699424 10.1371/journal.pone.0093395 PMC3974748

[23] Muradrasoli S, Bálint Á, Wahlgren J, et al Prevalence and phylogeny of coronaviruses in wild birds from the Bering Strait area (Beringia). PLoS One. 2010;5:e13640. 21060827 10.1371/journal.pone.0013640 PMC2966397

[24] Chu DK, Leung CY, Gilbert M, et al Avian coronavirus in wild aquatic birds. J Virol. 2011;85:12815–12820. 21957308 10.1128/JVI.05838-11 PMC3209365

[25] Lindh E Avian influenza and newcastle disease viruses in Finland – genetics, epidemiology and ecology in the natural host, wild waterfowl [Ph.D. thesis]. Helsinki: Faculty of Medicine, University of Helsinki; 2015.

[26] Muradrasoli S, Mohamed N, Hornyák A, et al Broadly targeted multiprobe QPCR for detection of coronaviruses: coronavirus is common among mallard ducks (Anas platyrhynchos). J Virol Methods. 2009;159:277–287. 19406168 10.1016/j.jviromet.2009.04.022 PMC7112901

[27] Larkin MA, Blackshields G, Brown NP, et al Clustal W and Clustal X version 2.0. Bioinformatics. 2007;23:2947–2948. 17846036 10.1093/bioinformatics/btm404

[28] Hall TA BioEdit: a user-friendly biological sequence alignment editor and analysis program for Windows 95/98/NT. Nucl Acids Symp Ser. 1999;41:95–98.

[29] Tamura K, Stecher G, Peterson D, et al MEGA6: molecular evolutionary genetics analysis version 6.0. Mol Biol Evol. 2013;30:2725–2729. 24132122 10.1093/molbev/mst197 PMC3840312

[30] The Basic Local Alignment Search Tool (BLAST) [cited 2016 7 3]. Available from: http://blast.ncbi.nlm.nih.gov/Blast.cgi

[31] GenBank NCBI [cited 2016 7 3] Available from: http://www.ncbi.nlm.nih.gov/genbank/

[32] Lindh E, Ek-Kommonen C, Väänänen V-M, et al Molecular epidemiology of H9N2 influenza viruses in Northern Europe. Vet Microbiol. 2014;172:548–554. 25042528 10.1016/j.vetmic.2014.06.020

[33] Zhuang Q-Y, Wang K-C, Liu S, et al Genomic analysis and surveillance of the coronavirus dominant in ducks in China. PLoS One. 2015;10:e0129256. 26053682 10.1371/journal.pone.0129256 PMC4459809

[34] Kim H-R, Oem J-K Surveillance of avian coronaviruses in wild bird populations of Korea. J Wildl Dis. 2014;50:964–968. 24949927 10.7589/2013-11-298

[35] Wille M, Avril A, Tolf C, et al Temporal dynamics, diversity, and interplay in three components of the virodiversity of a Mallard population: influenza A virus, avian paramyxovirus and avian coronavirus. Infect Genet Evol. 2015;29:129–137. 25461850 10.1016/j.meegid.2014.11.014 PMC7106038

[36] Tolf C, Wille M, Haidar A-K, et al Prevalence of avian paramyxovirus type 1 in Mallards during autumn migration in the western Baltic Sea region. Virol J. 2013;10:285-422X-10-285. 10.1186/1743-422X-10-285 PMC3847450 24028398

[37] Wille M, Muradrasoli S, Nilsson A, et al High prevalence and putative lineage maintenance of avian coronaviruses in scandinavian waterfowl. PLoS One. 2016;11:e0150198. 26938459 10.1371/journal.pone.0150198 PMC4777420

[38] Jonassen CM, Kofstad T, Larsen I-L, et al Molecular identification and characterization of novel coronaviruses infecting graylag geese (Anser anser), feral pigeons (Columbia livia) and mallards (Anas platyrhynchos). J Gen Virol. 2005;86:1597–1607. 15914837 10.1099/vir.0.80927-0

[39] Hughes LA, Savage C, Naylor C, et al Genetically diverse coronaviruses in wild bird populations of northern England. Emerg Infect Dis. 2009;15:1091–1094. 19624927 10.3201/eid1507.090067 PMC2744231

