# Supplementary material for: Prevalence and genetic diversity of coronaviruses in wild birds, Finland
Source: Infect Ecol Epidemiol. 2017 Nov 28;7(1):1408360. doi: 10.1080/20008686.2017.1408360 (PMC6369310; doi:10.1080/20008686.2017.1408360)
Supplement: Manuscript1Suppl.doc [file ZIEE_A_1408360_SM2116.doc]

**Supplementary Table S1. Overview of the bird samples.** A table indicating the 61 bird species and the number of representatives from each species included in the study.

| **Taxonomic family** | **Common name** | **Latin name** | **No. birds screened** |
| --- | --- | --- | --- |
| **Accipitridae** | Northern Goshawk | *Accipiter gentilis* | 49 |
|  | Eurasian Sparrowhawk | *Accipiter nisus* | 16 |
|  | Golden Eagle | *Aquila chrysaetos* | 16 |
|  | Common Buzzard | *Buteo buteo* | 2 |
|  | Rough-legged Buzzard | *Buteo lagopus* | 4 |
|  | Western Marsh Harrier | *Circus aeruginosus* | 2 |
|  | White-tailed Eagle | *Haliaeetus albicilla* | 49 |
| **Alcidae** | Razorbill | *Alca torda* | 17 |
|  | Common Murre | *Uria Aalge* | 3 |
| **Anatidae** | Northern Pintail | *Anas acuta* | 6 |
|  | Northern Shoveler | *Anas clypeata* | 1 |
|  | Eurasian Teal | *Anas crecca* | 55 |
|  | Eurasian Wigeon | *Anas penelope* | 23 |
|  | Mallard | *Anas platyrhynchos* | 129 |
|  | Greylag Goose | *Anser anser* | 1 |
|  | Bean Goose | *Anser fabalis* | 2 |
|  | Tufted Duck | *Aythya fuligula* | 1 |
|  | Canada Goose | *Branta canadensis* | 5 |
|  | Barnacle Goose | *Branta leucopsis* | 6 |
|  | Common Goldeneye | *Bucephala clangula* | 17 |
|  | Long-tailed Duck | *Clangula hyemalis* | 8 |
|  | Whooper Swan | *Cygnus cygnus* | 78 |
|  | Mute Swan | *Cygnus olor* | 2 |
|  | Common Scoter | *Melanitta nigra* | 1 |
|  | Common Merganser | *Mergus merganser* | 12 |
|  | Common Eider | *Somateria mollissima* | 10 |
| **Columbidae** | Pigeon | *Columba sp.* | 56 |
| **Corvidae** | Common Raven | *Corvus corax* | 3 |
|  | Carrion Crow | *Corvus corone* | 13 |
|  | Western Jackdaw | *Corvus monedula* | 6 |
|  | Eurasian Magpie | *Pica pica* | 21 |
| **Falconidae** | Peregrine Falcon | *Falco peregrinus* | 1 |
|  | Kestrel | *Falco tinnunculus* | 2 |
| **Gaviidae** | Black-throated Loon | *Gavia arctica* | 10 |
|  | Red-throated Loon | *Gavia stellata* | 6 |
| **Gruidae** | Common Crane | *Grus grus* | 14 |
| **Haematopodidae** | Eurasian Oystercatcher | *Haematopus ostralegus* | 1 |
| **Laridae** | European Herring Gull | *Larus argentatus* | 52 |
|  | Common Gull | *Larus canus* | 14 |
|  | Lesser Black-backed Gull | *Larus fuscus* | 12 |
|  | Black-headed Gull | *Chroicocephalus ridibundus* | 24 |
| **Pandionidae** | Common pheasant | *Phasianus colchicus* | 8 |
| **Paridae** | Eurasian Blue Tit | *Parus caeruleus* | 1 |
|  | Great Tit | *Parus major* | 1 |
| **Phalacrocoracidae** | Great Cormorant | *Phalacrocorax carbo* | 4 |
| **Phasianidae** | Hazel Grouse | *Bonasa bonasia* | 30 |
|  | Willow Ptarmigan | *Lagopus lagopus* | 6 |
|  | Common Pheasant | *Phasianus colchicus* | 1 |
|  | Black Grouse | *Tetrao tetrix* | 18 |
|  | Western Capercaillie | *Tetrao urogallus* | 17 |
| **Picidae** | White-backed Woodpecker | *Dendrocopos leucotos* | 2 |
|  | Great spotted Woodpecker | *Dendrocopos major* | 2 |
| **Strigidae** | Boreal Owl | *Aegolius funereus* | 3 |
|  | Short-eared Owl | *Asio flammeus* | 2 |
|  | Long-eared Owl | *Asio otus* | 8 |
|  | Eurasian Eagle-owl | *Bubo bubo* | 26 |
|  | Eurasian Pygmy owl | *Glaucidium passerinum* | 4 |
|  | Tawny Owl | *Strix aluco* | 6 |
|  | Great Grey Owl | *Strix nebulosa* | 15 |
|  | Ural Owl | *Strix uralensis* | 30 |
| **Strigidae** | Northern Hawk-owl | *Surnia ulula* | 5 |
|  |  |  | 939 |
